# The durability of examination gloves used on intensive care units

**DOI:** 10.1186/1471-2334-13-226

**Published:** 2013-05-20

**Authors:** Nils-Olaf Hübner, Anna-Maria Goerdt, Axel Mannerow, Ute Pohrt, Claus-Dieter Heidecke, Axel Kramer, Lars Ivo Partecke

**Affiliations:** 1 Institute of Hygiene and Environmental Medicine, Ernst-Moritz-Arndt University, Greifswald, Germany; 2 Robert Koch-Institute, Division of Applied General and Hospital Hygiene (FG14), Berlin, Germany; 3 Department of Surgery, Clinic of General, Visceral, Vascular and Thoracic Surgery, Greifswald, Germany; 4 German Social Accident Insurance Institution for the Health and Welfare Services, Berlin, Germany

**Keywords:** Hand hygiene, Examination gloves, Micro perforation, Glove change, Intensive care unit, Disinfection, Multi-barrier strategy

## Abstract

**Background:**

The use of examination gloves is part of the standard precautions to prevent medical staff from transmission of infectious agents between patients. Gloves also protect the staff from infectious agents originating from patients. Adequate protection, however, depends on intact gloves. The risk of perforation of examination gloves is thought to correlate with duration of wearing, yet, only very few prospective studies have been performed on this issue.

**Methods:**

A total number of 1500 consecutively used pairs of examination gloves of two different brands and materials (latex and nitrile) were collected over a period of two months on two ICU’s. Used gloves were examined for micro perforations using the “water-proof-test” according to EN 455–1. Cox-regression for both glove types was used to estimate optimal changing intervals.

**Results:**

Only 26% of gloves were worn longer than 15 min. The total perforation rate was 10.3% with significant differences and deterioration of integrity of gloves between brands (p<0.001). Apart from the brand, “change of wound dressing” (p = 0.049) and “washing patients” (p = 0.001) were also significantly associated with an increased risk of perforation.

**Conclusion:**

Medical gloves show marked differences in their durability that cannot be predicted based on the technical data routinely provided by the manufacturer. Based on the increase of micro perforations over time and the wearing behavior, recommendations for maximum wearing time of gloves should be given. Changing of gloves after 15 min could be a good compromise between feasibility and safety. HCWs should be aware of the benefits and limitations of medical gloves. To improve personal hygiene hand disinfection should be further encouraged.

## Background

Hospital acquired infections (HAIs), caused for example by multi-drug resistant organisms as Methicillin-resistant *Staphylococcus aureus* or spore-forming bacteria like Clostridium difficile, are a severe menace particularly on intensive care units (ICU) [1]. The hands of the hospital personnel have been acknowledged as the most important transmission route of these pathogens [2,3]. Hand hygiene plays therefore a key role for the prevention of infections. Apart from hand disinfection, wearing gloves is an essential part of the multi-barrier strategy for hand hygiene.

Gloves function as mechanical barriers that help to reduce transmission of body fluids and pathogens from patients to hospital personnel and vice versa. They effectively reduce the contamination of health care worker’s (HCW) hands and protect the hospital staff from viral infections like HIV and hepatitis. These functions depend on to the integrity of the glove and/or the absence of perforations of the glove. Recent studies with surgical gloves demonstrated that the frequency of micro perforations is significantly correlated with the duration of glove wearing [4-6]. Different factors may influence the integrity of the glove such as the material [7], the wearing time, the activities, the fit and the brand [7]. Muto et al. described a higher frequency of micro perforations in examination gloves than in surgical gloves [8].

Despite the fact that the barrier function of gloves primarily depends on intact material the migration of infectious agents through unnoticed micro perforations has been demonstrated [9]. Therefore, specific recommendations for the time point when to change examination gloves while working with patients on intensive care units are required.

This study evaluated the frequency of unnoticed micro perforations in two different types of examination gloves in relation to the duration of wearing. Based on these results we would like to provide evidence based recommendations for glove changing during work on ICUs.

## Methods

During a consecutive period of two months (April and May 2008) all worn examination gloves on two intensive care units (unit 1: 7 beds, unit 2: 11 beds) of the University Medicine, Greifswald, Germany, were prospectively collected and examined.

The study did not in any way interfere with human subjects or medical treatment, but was a purely observational asessment of material behaviour of gloves as part of personal protective equipment (PPE) unter real world conditions. Therefore, no formal approval by an ethics committee nor working council was required under German regulations.

Utilized gloves included latex gloves (Glove brand A, GBA; PehaSoft^®^Powderfree, Paul Hartmann AG, Heidenheim, Germany) and nitrile gloves (Glove brand B, GBB; Nitra-Tex^®^EP Powderfree, Ansell, Brussels, Belgium), in the standard sizes Small, Medium and Large. All gloves fulfilled the requirements of EN 455–2 for unsterile medical single-use gloves and complied with an Acceptance Quality Limit (AQL) of <1.5. The gloves were examined for micro perforations using the water impermeability test according to DIN EN 455–1 as previously described [4,10].

Type of material (latex or nitrile), duration of wearing (minutes), type of procedure the gloves were used for (i.e. washing patients, change of dressings, help with food, application of medication or taking blood samples), contact to disinfectants (skin, hand, surface), general care procedures, fitting of gloves (accurately vs. unsuitable fitting, i.e. too tight or loose-fitting) and the person who wore the gloves were recorded directly after removing the glove, described by anonymous selfreporting by the user. As our study was purely observational, reporting of more than one task performed with the same pair of gloves was possible to minimize any observer effects.

An alcohol based hand rub (Softa-Man®, B. Braun, Melsungen, Germany) was exclusively used throughout this study. It contained 45% ethanol and 18% 1-propanol and is approved for both hygienic and pre-surgical hand disinfection.

To assess additional effects of mechanical stress and duration of wearing on the perforation rate in particular, 50 pairs of brand new gloves as well as 50 pairs of GBB and GBA examination gloves were analyzed as control group worn by students for 60 minutes during a lecture.

### Statistical analysis

A Cox-regression for both glove types was used to estimate optimal changing intervals, defining fitting of gloves (fitting/non-fitting), wearer and the procedures performed (change of dressing” (yes/no), washing patients”(yes/no), general duties (yes/no), medicines and blood sampling (yes/no), help with eating (yes/no), contact to disinfectants (yes/no) as covariates.

SPSS 20 (SPSS Chicago Inc.) and Prism 5 (GraphPad Software Inc., San Diego, California) were used for data analysis and illustrations. A difference was considered as statistically significant if p was <0.05.

## Results

**Figure 1:**
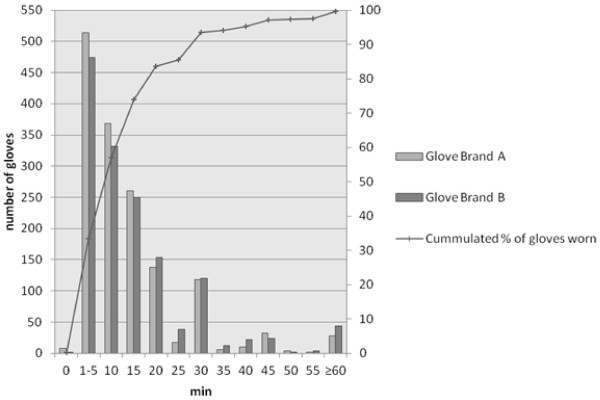
**Distribution of wearing times for both glove brands and cumulated number of total gloves worn up to a set time.** The distribution is very positively skewed with only 26% of gloves worn longer than 15 minutes and almost 94% worn within 30 minutes.

A total of 3000 (1508 GBA and 1482 GBB) examination gloves (1500 pairs) were collected and examined. Of all participating HCW (n = 58) 55 were right-handed and 3 left-handed, respectively. The majority of HCWs were nurses. Most gloves (56.9%) were worn for up to 10 minutes. Interestingly, the mean wearing time of all tested gloves was longer (14 minutes, range 1 to 135 minutes). This difference was caused by a relatively small fraction of gloves with extremely long wearing times compared to the majority of gloves (Figure 1). A subgroup analysis of the glove material revealed only marginal differences in mean wearing times of both glove types with GBB worn 14.8 minutes [CI95: 14.1 - 15.4] and GBA worn 13.7 minutes [CI95: 13.0 to 14.3].

**Figure 2:**
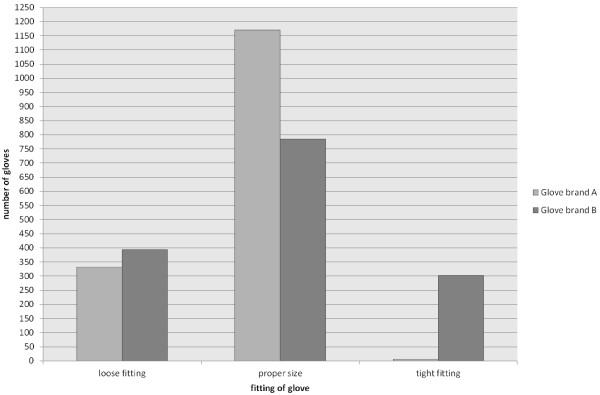
**Fitting of gloves.** Most gloves were reported to have the proper size, but loose or tight fitting was a relevant issue, especially for the less flexible nitrile gloves.

Most gloves were reported to have the proper size, but loose or tight fitting was a relevant issue (34.6% overall), especially for the less flexible nitrile gloves (Figure 2).

A total of 308/3000 (10.3%) of gloves were perforated. In only 5.2% of cases, perforations were noticed by the HCWs. In 308 gloves identified as being perforated, 389 perforations were detected (1.26 perforations per glove on average). Crude perforation rates of 9.0% (10 minutes), 15.6% (20 minutes), 27.2% (30 minutes), 30.5% (50 minutes), 54.2% (60 minutes) and 50.0% (>61 minutes) for GBA and 5.1% (10 minutes), 5.4% (20 min), 10.8% (30 minutes), 23.1% (50 minutes), 17.4% (60 minutes) and 16.7% (>61 minutes) for GBB were observed.

Locations of micro perforations of all tested gloves were as follows: thumb 23.7%, palm 18.8%, cuff 17.7% at the index finger 17.2%, middle finger 11.8%, pinky 4.1%, and with the lowest perforation rates at the ring finger (3.6%) and inter digitally (3.6%).

**Figure 3:**
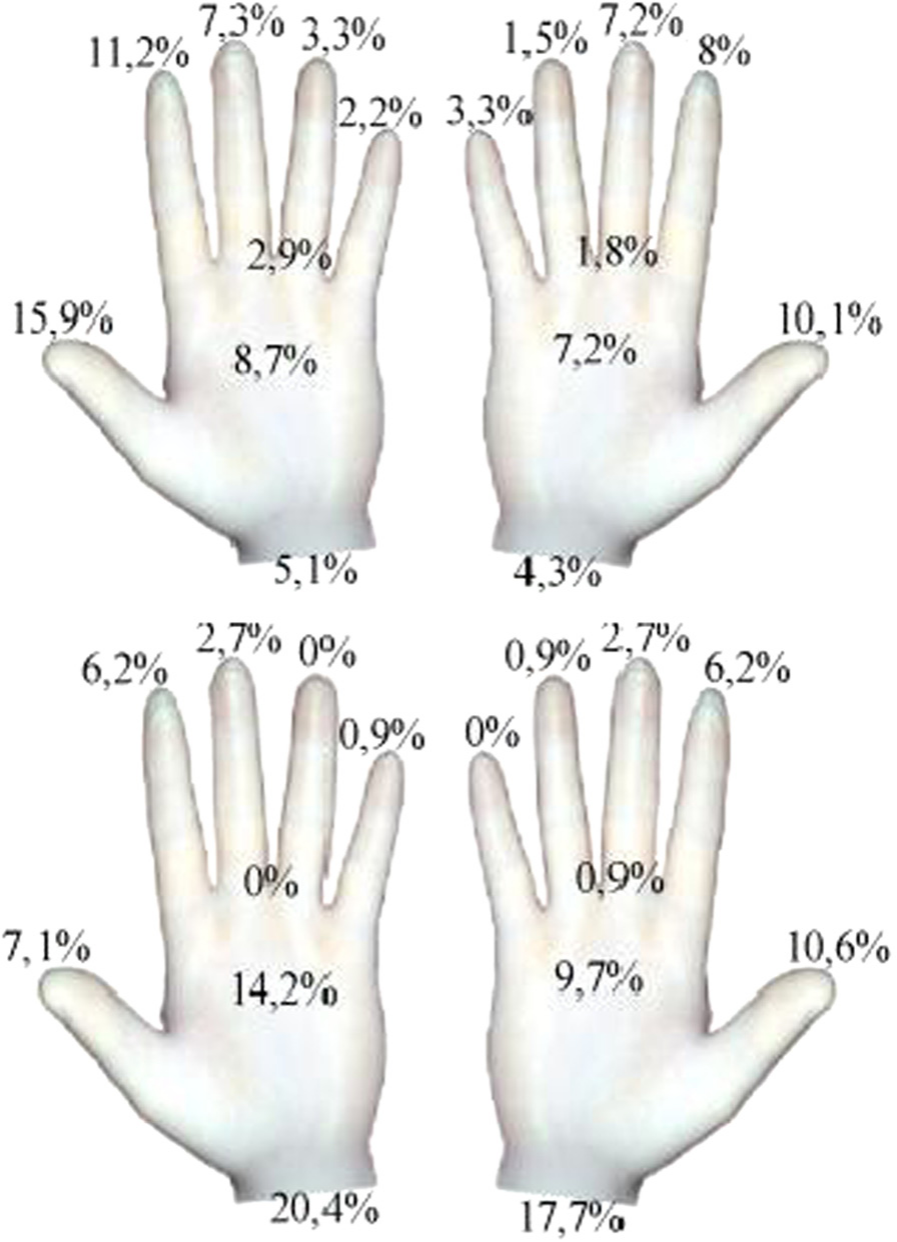
**Distribution of micro perforations of both brands.** Brand A gloves (upper illustration) were mostly perforated at the fingers (thumb and index finger), while brand B gloves (lower illustration) were more often perforated at on cuffs and palms.

Subgroup analyses revealed that the frequency and distribution of perforations differed notably between GBA and GBB (Figure 3).

While the thumb (26.0%) and the index finger (19.2%) were most often affected in GBA, the cuff (38.1%) and the palm 23.9% were most often perforated in GBB. 210 of 1508 (13.9%) GBA showed micro perforations with a total number of 276 perforations detected (1.31 perforations per glove on average). In comparison, 98 of 1482 (6.6%) GBB were identified as perforated, with a total number of 113 perforations (1.15 perforations per glove on average).

To identify relevant factors for glove failure a Cox regression analysis was performed (Table 1). Glove failure was defined as one or more perforations per glove. In 9 cases data on use and/or wearer were missing and these were excluded from the analysis.

**Table 1:** Results from the Cox regression analysis

**Cofactor**	**p**	**Adjusted hazard ratio (aHR)**	**95.0% CI**	
			**Lower**	**Upper**
Wearer	0.141	1.008	0.998	1.018
Brand	0.001	2.498	1.927	3.238
Fitting	0.252	0.861	0.665	1.113
General duties	0.086	1.312	0.962	1.791
Giving Medicine and blood sampling	0.398	1.149	0.832	1.587
Help with eating	0.458	0.797	0.438	1.450
Change of dressing	0.049	1.498	1.002	2.240
Washing patients	0.001	1.737	1.248	2.416
Contact to disinfectants	0.084	1.259	0.969	1.634

**Figure 4:**
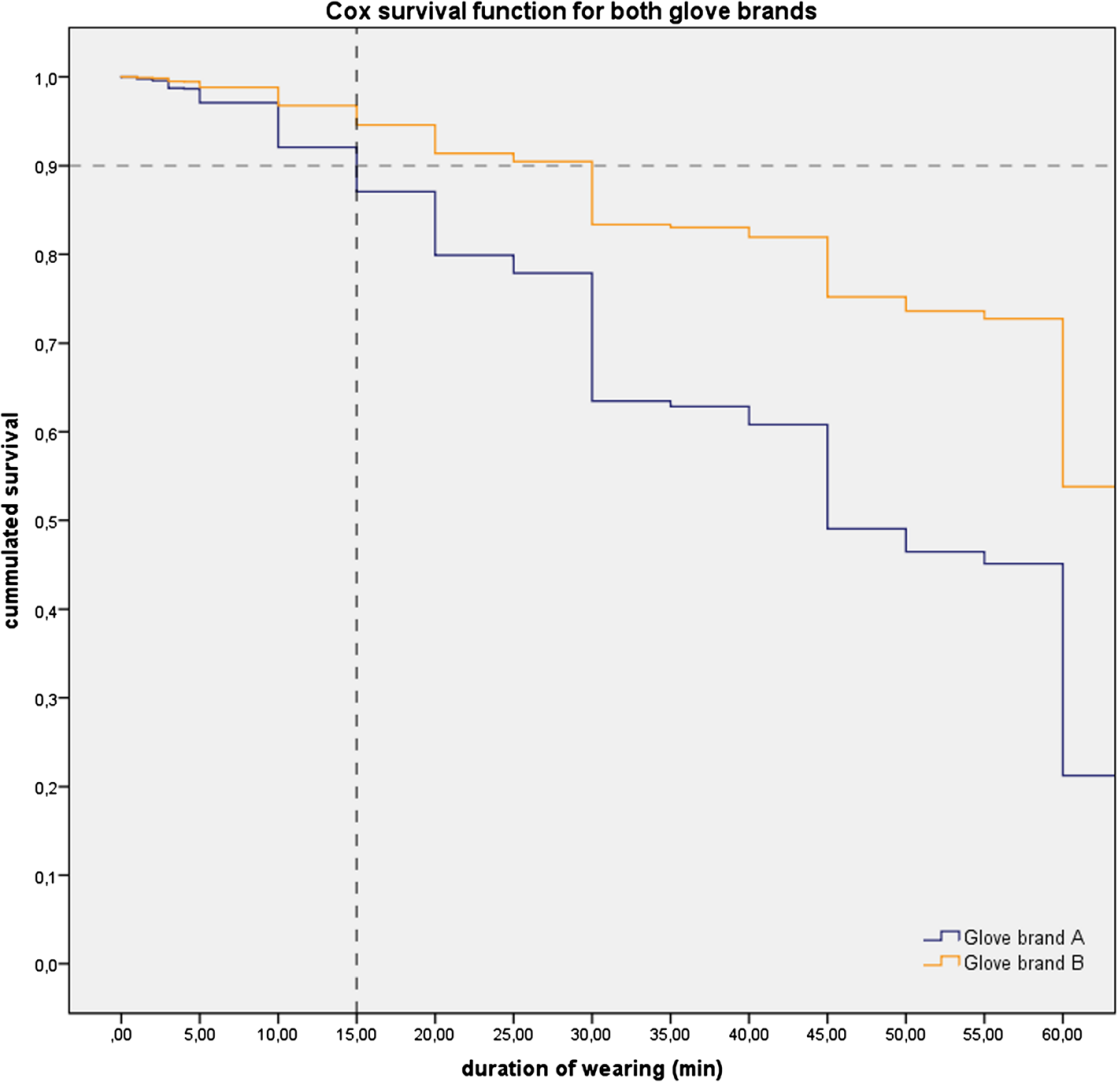
**Cox survival function for both glove brands.** Percentage of unperforated gloves drops below 90% after 15 min, with brand A showing a much steeper detioration curve than brand B.

The multivariate analysis revealed that perforation rates of the brands differed highly significantly (adjusted Hazard Ratio = 2.498, p = 0.001) with gloves of brand A showing a much steeper decrease in integrity than brand B (Figure 4).

Apart from the brand, “change of dressing” (adjusted Hazard Ratio = 1.489, p = 0.049) and “washing patients” (adjusted Hazard Ratio = 1.737, p = 0.001) were also significantly associated with an increased risk of perforation.

In the control group with minimal mechanical stress (students wearing gloves during a lecture) only one latex glove (2%) out of 50 and two nitrile gloves (4%) out of 50 showed micro perforations. No perforation was detected in brand-new nitrile gloves and one out of 100 brand-new latex gloves had a perforation located at the pinky. Both results are well within the perforation rate to be expected by the AQL of the gloves.

## Discussion

The importance of nosocomial transmission of infective agents on ICUs cannot be overemphasized [11,12].

The key role of HCWs hands in the transmission of pathogens from patient to patient was demonstrated more than 150 years ago by Ignaz Semmelweis [13] and hand hygiene is recognized as key factor for infection prevention [14]. Still hand-hygiene compliance of HCWs is often unacceptably low [15]. Glove use, particularly on intensive care units, can add an additional security layer and substantially improve the so-called multi-barrier concept of hygiene. Kuzu et al. could demonstrate that compliance with glove use is relatively high [16].

Gloves should be used to protect from any anticipated contact with blood, mucous membranes, non-intact skin, secretions, and body fluids of all patients [17]. The preventive effect depends largely on the integrity of the glove. However, gloves may perforate unnoticed or may tear during use so hands can become contaminated [4,9,18-21]. Doebbeling et al. [22] showed that 5 to 10% of hands of HCW’s were contaminated after glove removal. This shows that occult micro perforations in gloves may play an important role for cross-transmission of pathogens if hands are not thoroughly disinfected before gloving and after degloving [23,24]. Previously, we have demonstrated that microorganisms do in fact penetrate through micro perforations under working conditions stressing the need for an intact glove to maintain the barrier [9,18]. This phenomenon is of particular concern on ICUs where patient care requires frequent HCW to patient contact and a high workload is common.

To the best of our knowledge, this is the first observational study that investigated the duration of wearing of gloves by HCWs on ICUs and the rate of associated micro perforations in gloves. The results of this study and evidence from literature indicate that the occurrence of micro perforations and consequent loss of the protective barrier function increases with the duration of wearing, stressing the need for thorough hand disinfection and challenging current recommendations that do not limit duration of glove use.

Our data indicate that not mechanical stress of putting on or handling over the glove or the time of wearing alone without interspace, but the combination with HCWs tasks is the cause of micro perforations in gloves. While some procedures seem to cause more damage to the integrity of the glove than others, all procedures are part of the routine tasks on a ward. The order of these procedures changes randomly and is not planable.

Thus, choosing the right type of glove that can resist the stress of use as well as frequent glove changes is crucial in order to minimize the risk of undetected micro perforations and potential transmission of infectious agents.

Both glove brands used in this study comply with the requirements of medical gloves. The differences between both glove brands are therefore striking and show that the user cannot predict the durability of gloves based on the technical data provided by the manufacturer. Not only the perforation rate but also the localisation of micro perforations showed notable differences between both brands. Brand A gloves were mostly perforated at the fingers (thumb and index finger), whereas brand B gloves showed most perforations at the cuffs and the palms (Figure 3). This is remarkable, since GBA were a little thicker as GBB (GBA: 0.200/0.130/0.11 GBB: 0.155/0.120/0.100 [Finger/Palm/Cuff]) according to technical datasheets provided by the manufacturers. In contrast to our previous data on surgical gloves showing most perforations at the non-dominant hand, mainly at the index finger [4], this study could show that perforations of examination gloves were almost equally distributed over the fingers and the palm of both hands, indicating different risk factors for perforations of both glove types and associated use. Several tasks in patient care imply extreme mechanical and/or chemical stress for gloves and bear a particular risk of pathogen transmission. While washing a patient, for example, the glove gets in contact with detergents and cosmetic additives and is exposed to mechanical stress when rubbing or moving a patient. The humid milieu while washing a patient additionally favours a translocation of microorganisms. Defining “risky” and “safe” procedures, however, is misleading and could result in a false feeling of security. Moreover, a certain percentage of medical gloves can be expected to have microperforations right out of the box in a frequency that depends on the AQL. Regular change of gloves and hand disinfection before and after glove use are therefore indicated.

For defining the optimal changing time the two factors “safety and feasibility” should be considered. Safety in this respect would mean the number of micro perforations to be expected and feasibility the number of procedures where a change in behavior of changing gloves would be needed if a maximum wearing time is set, since too frequent glove changes could lower the compliance with hygienic measures and waste time of HCWs and financial resources. Therefore, an optimal interval of glove changing has to be estimated, based on the wearing behavior and the time-dependent rate of perforations. Figure 1 shows the distribution of times the gloves were worn. Only 26% of gloves were worn longer than 15 minutes. After this time, on the other hand, the survival rate of both brands drops below 90% (Figure 4), meaning a threefold increase in the perforation rates in comparison to the AQL. Based on our data 15 minutes seems to be a pragmatic recommendation for regular glove changing, but we do not believe our data to be sufficient for a general recommendation for a specific time for routine glove changing, since too many factors do influence the perforation rate. Nonetheless, our study showed that such recommendations should be given in general and HCWs should acknowledge the role of micro perforations and the correlation of micro perforations with wearing time and certain activities. Health care providers, therefore, should define their own recommendations based on individual risk and feasibility assessments.

However, our study has several limitations. First, with the method used in this study we cannot determine the exact moment of perforation but differentiate defective from non-defective gloves after use only. Hence our estimate of the time dependency of the perforation rate is a very conservative one. In daily working routine the time of absent perforations may be much shorter.

The second limitation is that only two brands made of different materials were used. This is based on the central buying of the University Hospital of Greifswald supplying the complete University Medicine Greifswald with only two types of gloves. GBB showed a significantly lower perforation rate (6.6% vs. 13.9%) and should therefore be preferred to GBA. This result, however, is specific for these brands. While it shows the striking differences in the durability of individual brands, it cannot be generalized to the material or to the manufacturer of the gloves. Moreover, our results aim at the work of HCWs on specialised ICU settings and should not be generalised to different settings like paramedics or laboratories. For these settings, further studies would be necessary. Also, using different types of procedures on one glove could lead to different risks of micro perforations. Finally, since the number of examined gloves rapidly decreased for times over 30 minutes (only 6.4% were worn longer than 30 minutes) perforation rates for longer times should be interpreted with great care as they are based on small numbers.

However, our results are in line with results from other studies. Pitten et al. have already shown the influence of disinfectants on the rate of micro perforations in examination gloves depending on the brand [25] and that remarkable differences existed in the durability of gloves under routine working conditions [26]. While for 68.8% of gloves contact to disinfectants (skin, hand, surface) during use was reported, our data showed no significant effect on the perforation rate for these brands in the Cox regression analyses (B=0.232, p=0.083).

## Conclusions

While our data do not allow to draw general conclusions about the optimal changing interval or type of material that is preferable for medical gloves, we were able to show that micro perforations and poor fitting of gloves are a common problem with examination gloves. Also the integrity of gloves of different brands and materials deteriorated during intended use. Moreover, the user cannot predict the durability of gloves based on the technical data provided by the manufacturer.

Further research should be focused on providing the user with information to predict the durability of gloves as well as optimizing durability and fitting of gloves.

HCWs should be aware of the benefits and limitations of medical gloves and compliance with hand disinfection should be encouraged.

## Competing interests

The authors declare that they have no competing interest.

## Authors’ contribution

NOH, LIP, AM and AK and CDH designed the study, AM examind the gloves and collected the data, UP, NOH and AM analysed the data, AMG, NOH and LIP wrote the manuscript and all authors read, revised and approved the final manuscript.

## Pre-publication history

The pre-publication history for this paper can be accessed here:

http://www.biomedcentral.com/1471-2334/13/226/prepub
